# Seen and Heard in Many Places

**Published:** 1897-04

**Authors:** 


### SEEN AND HEARD IN MANY PLACES.

The New York State Veterinary College has succeeded in getting in the State supply-bill an item of $30,000 for maintenance. This assures the school continued support by the State.

Veterinary Surgeon Desmond, of Warrnambool, Australia, has a territory of two hundred and fifty miles to cover. His nearest colleague was one hundred and twenty miles away. Surely there was little chance for mingling views with his fellow-practitioner, and it must have been long waits between consultations.

The commencement exercises of the Kansas City Veterinary College this year will partake of a social character rather than a public function. The conferring of the degrees will be followed by a banquet.

The Atlantic Transport Line from New York, now engaged in seeking the foreign shipment trade in horses, has established a veterinary service corps in connection therewith. Each vessel has a veterinarian attached to the staff of officers, and all sickness and accidents among the horses are given professional attention. This is a wise movement.

The Milk Department of the Philadelphia City Board of Health in it annual report comes out strongly against the sale of milk in sealed jars and glass vessels.

The Buffalo *Horse World* inveighs strongly against the present law of the Empire State regarding the practice of the shoeing craft. The opposition is caused by the fact that one of their shoers was so indifferent to the existence of the law regulating the craft that he failed to register under its provisions. He subsequently presented himself, but failed in the examinations.

Maine has on hand a serious case involving the sale by one of her city butchers of a lot of cattle which had been condemned as tuberculous and ordered to be placed in the rendering-tank. Such daring effrontery as this should be most severely punished.

Practice in Iowa is reported by one of our correspondents as better in some localities, but throughout the State generally quiet.

In Mexico, turkeys, like sheep, are driven to market through the main streets.

A baby elephant in the winter-quarters of Dr. W. A. Conklin, in Brooklyn, having contracted a cold, was given quinine and whiskey for the same. He then gained access to the source of supply, became intoxicated, and encountered a huge phyton, which ended in the death of both elephant and snake.

The Poland China boar hog Happy Union brought the phenomenal price of $4000 at a sale in March at Jefferson, Iowa. He was bought by a syndicate of raisers. This sounds like the fabulous sums paid for standard-bred horses a few years ago, and may end in a grand crash, just as those prices in equine stock did, and from which recovery is very slow.

The Secretary of the Percheron Horse-breeders' Association gives as an additional reason for docking the sanitary proposition that it is an aid toward cleanliness.

"Ben," a venerable carriage horse at Long Branch, New Jersey, died recently. He was buried in state, and a tombstone was erected to his memory. He was forty-two years old.

In Paris they first utilize rats to clear the flesh from the bones of carcasses, then kill the rats, use their fur for trimmings, their skin for gloves, their thigh bones for toothpicks, and their tendons and bones for gelatin wrappers.

The owner of the stallion Allerton requires the following qualifications for mares to be stinted to this horse: They must be fashionably bred; their color must be bay, brown, black, or chestnut, with dark manes and tails; each mare must be from 15.1 to 16 hands high; all must be sound, stand square on their feet, and neither toe-in nor toe-out. They must all be square-gaited trotters (pacing mares by pacing horses will not be accepted). The limit of age will run from three to fifteen years. A mare over thirteen years old must have produced a fast trotter or have a fast trotting record herself. It will be interesting to watch the outcome of this wise step.

A recent shipment of horses from Nebraska to the vicinity of Philadelphia proved to have at least three cases of glanders in the lot. The balance of the shipment having been sold at a dispersal sale, their condition is not traced.

In a shipment of horses from the West to Boston a number of cases of glanders were discovered. Is it not time for us to have some interstate equine inspection?

